# Micellar Assembly and Disassembly of Organoselenium Block Copolymers through Alkylation and Dealkylation Processes

**DOI:** 10.3390/polym13152456

**Published:** 2021-07-27

**Authors:** Taejun Eom, Anzar Khan

**Affiliations:** Department of Chemical and Biological Engineering, Korea University, 145 Anam-Ro, Seongbuk-Gu, Seoul 02841, Korea; eomtorr@korea.ac.kr

**Keywords:** organoselenium polymers, selenium-epoxy ‘click’ reaction, alkylation/dealkylation, polymer assembly, micellar nanostructures, micellar disassembly

## Abstract

The aim of this work is to demonstrate that the alkylation and dealkylation of selenium atoms is an effective tool in controlling polymer amphiphilicity and, hence, its assembly and disassembly process in water. To establish this concept, poly(ethylene glycol)-block-poly(glycidyl methacrylate) was prepared. A post-synthesis modification with phenyl selenolate through a base-catalyzed selenium-epoxy ‘click’ reaction then gave rise to the side-chain selenium-containing block copolymer with an amphiphilic character. This polymer assembled into micellar structures in water. However, silver tetrafluoroborate-promoted alkylation of the selenium atoms resulted in the formation of hydrophilic selenonium tetrafluoroborate salts. This enhancement in the chemical polarity of the second polymer block removed the amphiphilic character from the polymer chain and led to the disassembly of the micellar structures. This process could be reversed by restoring the original amphiphilic polymer character through the dealkylation of the cations.

## 1. Introduction

**Figure 1:**
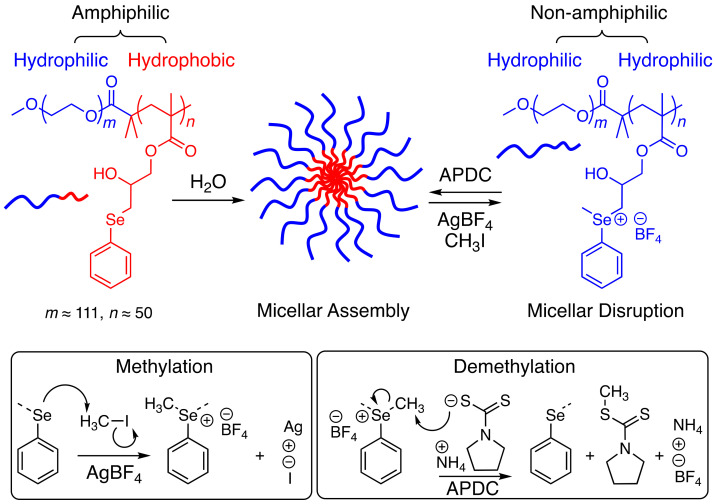
The concept of controlling chemical nature of the polymer chain and, thus, its assembly/disassembly behavior in water through alkylation/dealkylation process (APDC—ammonium pyrrolidinedithiocarbamate).

In nature, reversible methylation and demethylation processes play a critical role in determining protein structure and function [1]. In synthetic polymers, however, such reversible alkylation/dealkylation-based strategies are not employed in controlling polymer properties. Towards this end, we envisaged that the methylation of selenium (Se) atoms to selenonium (Se^+^) cations would result in a large change in the chemical polarity of the system. While organoselenides are neutral and hydrophobic, selenonium salts are positively charged and hydrophilic in nature. Therefore, if a copolymer chain contains a hydrophilic block and an organoselenium-based [2,3,4,5,6,7,8,9,10,11,12,13] hydrophobic block, it would assemble in water due to an amphiphilic nature of the polymer (Figure 1). However, if the selenium-containing polymer block is methylated, its chemical nature would change to reflect a hydrophilic character. Thus, upon alkylation, the amphiphilic character of the block copolymer would be lost and the micellar structure would be disrupted. If the alkylation and dealkylation can be carried out in a reversible fashion, the polymer micelles could be formed and broken through the methylation/demethylation of selenium atoms.

## 2. Results and Discussion

**Scheme 1:**
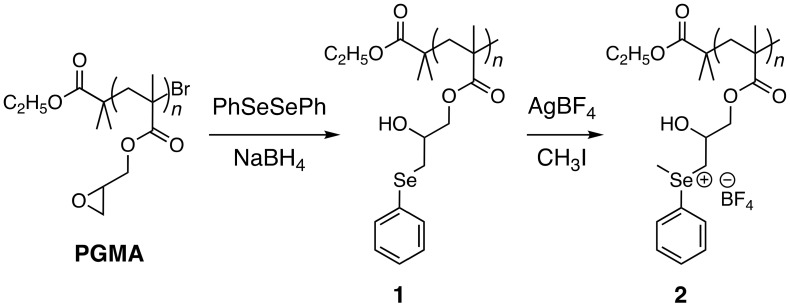
Synthesis of selenonium homopolymer **2**.

**Figure 2:**
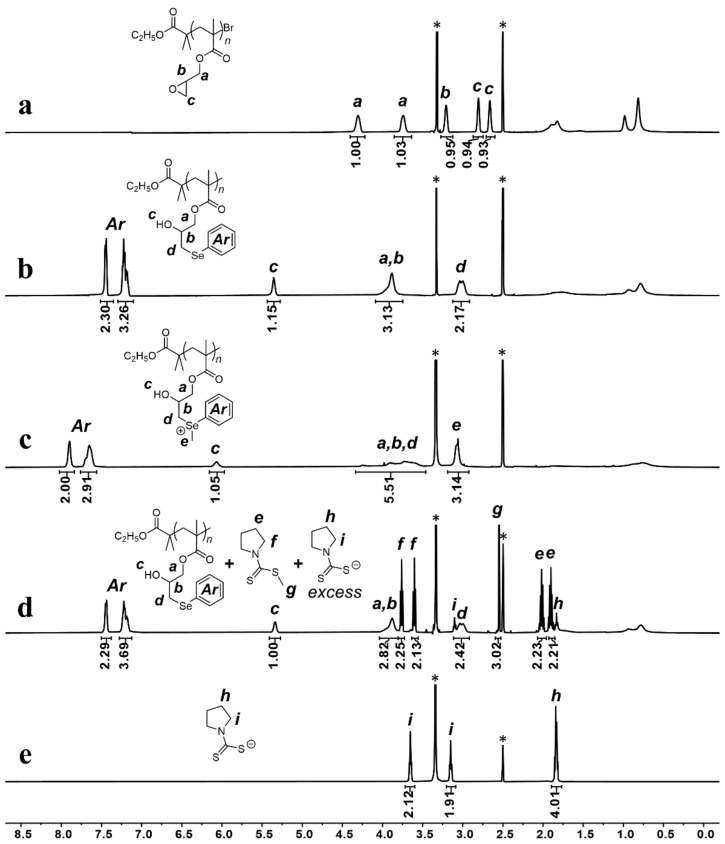
The ^1^H-NMR of PGMA (**a**), polymer **1** (**b**), and polymer **2** before (**c**) and after addition of a slight excess of APDC (1.1 equiv./cation) (**d**). The measurement was carried out immediately after the addition of APDC to the polymer solution. The bottom shows the ^1^H-NMR of APDC (**e**). Signals from residual solvents (water and dimethylsulfoxide) are shown with an asterisk.

To examine the feasibility of the aforementioned concept, it was necessary to first establish whether the selenonium polymer segment would be soluble and stable in water. To investigate these aspects, the epoxide side-chains of poly(glycidyl methacrylate) (*M*_n_ = 36,400, *M*_w_ = 41,900, *M*_w_/*M*_n_ = 1.15) were subjected to a base-catalyzed selenium-epoxy ‘click’ reaction to furnish polymer **one** (*M*_n_ = 42,500, *M*_w_ = 52,400, *M*_w_/*M*_n_ = 1.23) (Scheme 1, Figure 2 and Figure S1) [14,15]. The side-chain selenoethers could then be subjected to a methylation reaction using methyl iodide and silver tetrafluoroborate to give polymer **two** [14,15]. The use of silver tetrafluoroborate for the alkylation reactions is well established in small molecule and polymer chemistry [14,15,16,17]. The tetrafluoroborate anion is non-nucleophilic (in contrast to halide ions) and is known to enhance polycation stability [18]. Therefore, an ion exchange to chloride anion, as is typical in polyelectrolyte chemistry, was not pursued.

In ^1^H-NMR, the proton resonances from the methylene groups adjacent to the selenium atoms in polymer **one** were observed at 3 ppm (Figure 2). The aromatic signals were seen in the range of 7.2–7.5 ppm. Once the selenium atoms were methylated, they became electron-deficient. Therefore, the aromatic resonances shifted downfield to 7.5–8 ppm. Furthermore, the methyl signal appeared at 3.1 ppm in polymer **two**. Polymer **two** was found to be well soluble in water as all proton resonances could be clearly seen in deuterated water (Supplementary Materials, Figure S2). It was also found to be stable as no changes could be seen in the ^1^H-NMR spectrum upon prolonged storage in water at 37 °C.

Having access to **two** also enabled us to examine the demethylation process. For this, inspired by the studies of Deming and coworkers on sulfonium polymers, ammonium pyrrolidinedithiocarbamate (APDC) was used as the dealkylating agent [19]. APDC is a strong nucleophile that reverts the selenonium cations to their neutral selenoether form by removing the methyl group. In this process, the methyl group is transferred onto the nucleophile. This methyl group can be located in the ^1^H-NMR spectrum of the crude reaction mixture at 2.6 ppm. The aromatic resonances also shift completely up field, thus, indicating a quantitative removal of the alkyl groups from the selenium atoms. The demethylation reaction was found to be instantaneous as the crude reaction mixture was observed immediately upon the addition of APDC to the aqueous polymer solution.

**Scheme 2:**
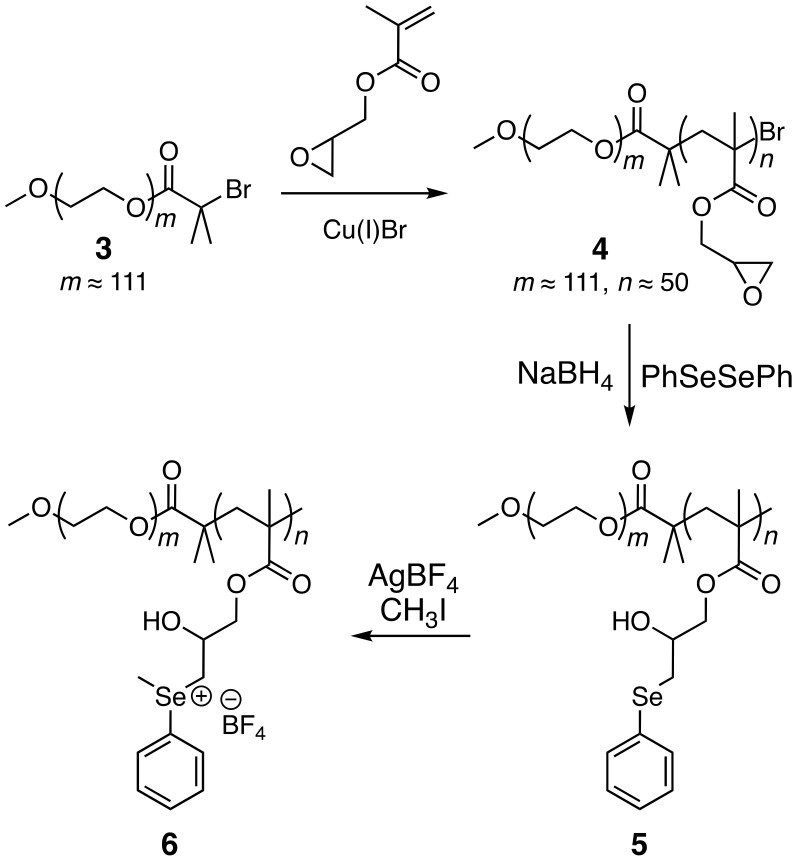
Synthesis of organoselenium block copolymers. The degree of polymerization was calculated with the help of area integration analysis in ^1^H-NMR spectroscopy.

Encouraged by these results, the synthesis of poly(ethylene glycol)-based block copolymer was targeted. For this, macroinitiator **three** was used to polymerize glycidyl methacrylate monomer through an atom transfer radical polymerization process [20]. This process led to the formation of reactive block copolymer **four** (*M*_n_ = 14,400, *M*_w_ = 17,900, *M*_w_/*M*_n_ = 1.24) (Scheme 2 and Figures S3 and S4). The sequence of ring-opening and methylation then gave access to polymers **five** (*M*_n_ = 16,300, *M*_w_ = 20,100, *M*_w_/*M*_n_ = 1.23) and **six** (Figure S5).

**Figure 3:**
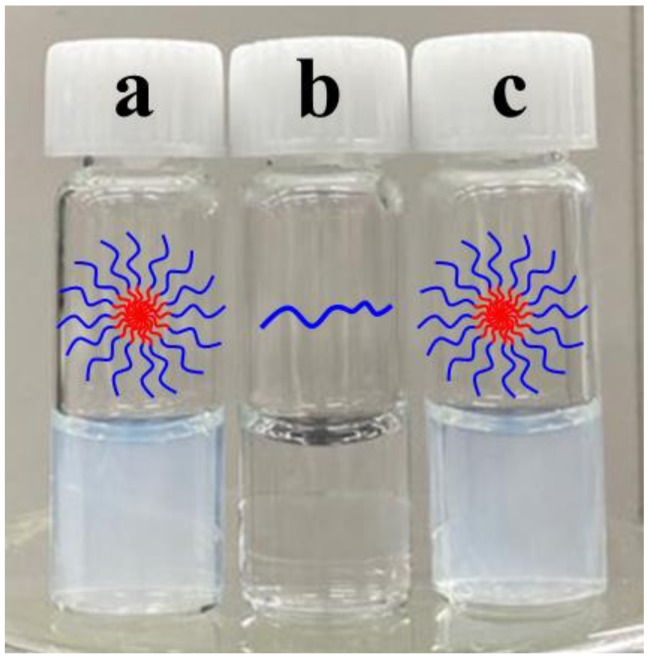
Digital photographs of aqueous solutions of polymer **5** (**a**), polymer **5** upon treatment with methyl iodide and silver tetrafluoroborate (**b**), and upon treatment with APDC (**c**).

**Figure 4:**
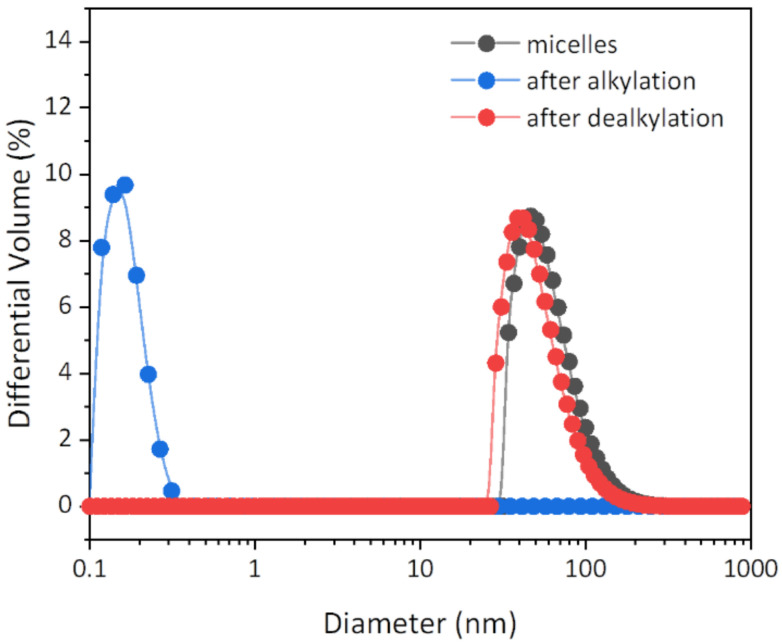
Dynamic light scattering analysis of the aqueous solution of polymer **5** before alkylation, after alkylation, and after dealkylation.

**Figure 5:**
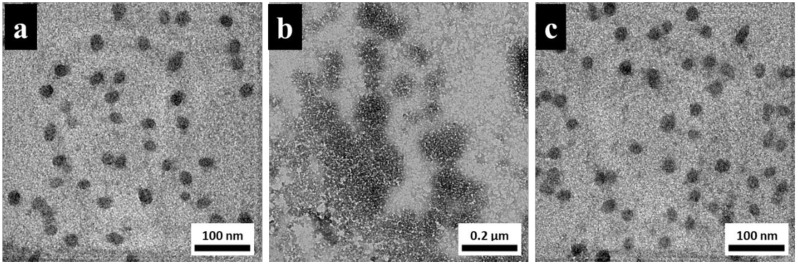
Transmission electron micrographs of samples prepared from an aqueous solution of polymer **5** before alkylation (**a**), after alkylation (**b**), and after dealkylation (**c**).

The poly(ethylene glycol) segment in polymer **five** is hydrophilic while the organoselenium segment is hydrophobic. Therefore, the overall character of the block copolymer is amphiphilic. The aqueous solutions of **5**, therefore, appeared to be cloudy due to the formation of micellar structures (Figure 3a). In this secondary structure, poly(ethylene glycol) segments formed a micellar shell and the organoselenium segment formed the micellar core. In this arrangement, the poly(ethylene glycol) chains maximized while the hydrophobic block minimized their interactions with water molecules. The nano-sized micellar structures were confirmed with the help of dynamic lighter scattering (DLS) (Figure 4) and transmission electron microscopy (TEM) (Figure 5a) analyses. DLS indicates micellar sizes of approximately 50–60 nm (Table S1). In TEM, the micellar cores higher in electron density due to the aromatic rings can be visualized with sizes in the range of 18–20 nm. Upon alkylation, however, the micellar assembly disrupted and in the DLS examination, a complete shift to unimolecular structures with sizes below 1 nm were seen. In TEM, an ill-defined large aggregate populated the substrate (Figure 5b). Visually, the aqueous solution appeared transparent due to the dissolution of the hydrophilic polymer chains in water (Figure 3b). This process can be reversed through in situ dealkylation with the help of APDC [19]. The demethylation was instantaneous in the case of block copolymers too (Figure S5) and led to the restoration of the originally coded amphiphilicity in the polymer structure and re-formation of the micellar assembly (Figure 4 and Figure 5c). Visually, the aqueous solution became cloudy once more, indicating the formation of nanostructures (Figure 3c). A control experiment indicated that the micelles remained unperturbed when subjected to the alkylation conditions in the absence of silver tetrafluoroborate and methyl iodide (Figure S6).

## 3. Conclusions

To summarize, the methylation of selenium atoms can convert an amphiphilic block copolymer into a hydrophilic structure through selenonium salt formation. Demethylation, on the other hand, can restore the original (chemically neutral) polymer character. Thus, while dealkylation facilitates the micellar assembly of the amphiphilic block copolymer in water, alkylation dissolves the assembled structure. Since methylating enzymes such as methyltransferases (MTases) are commonly found in cell biology, polymeric micelles sensitive to such natural cues can be useful for bio-relevant release applications.

## Data Availability

The data presented in this study are available in the article and the supplementary materials.

## Supplementary Materials

The following are available online at https://www.mdpi.com/article/10.3390/polym13152456/s1, Figure S1: GPC of PGMA (solid line) and polymer 1 (dash line), Figure S2: the ^1^H-NMR of polymer 2 in D_2_O at 37 °C. Residual solvent signal is shown with an asterisk, Figure S3: The ^1^H-NMR of polymer 4 in deuterated dimethylsulfoxide (DMSO). Residual solvent signal is shown with an asterisk, Figure S4: GPC of polymers 4 (solid line) and 5 (dash line), Figure S5: The ^1^H-NMR of polymer 5 (top), after alkylation (middle) and upon exposure with APDC (bottom). Signals from residual solvents are shown with an asterisk. The measurements were carried out immediately after addition of APDC to the polymer solution, Figure S6: DLS data for the control experiment in which the micelles of block copolymer 5 were exposed to 50 °C in absence of silver tetrafluoroborate and methyl iodide for 24 h prior to data acquisition (diameter = 63.1 nm, PDI = 0.196). Table S1: DLS data for polymer 5 before and after dealkylation process.
