# Supplementary material for: Micellar Assembly and Disassembly of Organoselenium Block Copolymers through Alkylation and Dealkylation Processes
Source: Polymers (Basel). 2021 Jul 27;13(15):2456. doi: 10.3390/polym13152456 (PMC8348486; doi:10.3390/polym13152456)
Supplement: Supplementary file 1 [file polymers-13-02456-s001.zip › polymers-1310491-supplementary.pdf]

Supplementary Material

Micellar Assembly and Disassembly of Organoselenium Block Copolymers through Alkylation and Dealkylation Processes

Taejun Eom and Anzar Khan *

Department of Chemical and Biological Engineering, Korea University, 145 Anam-Ro, Seongbuk-Gu, Seoul 02841, Korea; eomtorr@korea.ac.kr

* Correspondence: anzar@korea.ac.kr; Tel.: +82-2-3290-4859

Citation: Eom, T.; Khan, A. Micellar Assembly and Disassembly of Organoselenium Block Copolymers through Alkylation and Dealkylation Processes. *Polymers* **2021**, *13*, 2456. https://doi.org/10.3390/polym13152456

Academic Editor: Constantinos Tsitsilianis

Received: 7 July 2021

Accepted: 22 July 2021

Published: 27 July 2021

Publisher's Note: MDPI stays neutral with regard to jurisdictional claims in published maps and institutional affiliations.

Copyright: © 2021 by the authors. Licensee MDPI, Basel, Switzerland. This article is an open access article distributed under the terms and conditions of the Creative Commons Attribution (CC BY) license (http://creativecommons.org/licenses/by/4.0/).

General Methods and Materials

Glycidyl methacrylate, ethyl α-bromoisobutyrate (EBiB) 4,4′-dinonyl-2,2′-dipyridyl (dNbpy), Cu(I)Br, diphenyl diselenide, iodomethane, silver tetrafluoroborate ($AgBF_4$), sodium borohydride ($NaBH_4$), and ammonium pyrrolidinedithiocarbamate (APDC) were purchased from commercial sources. PEG macroinitiator (5kDa) was prepared according to the procedure described in *Polymer chemistry*, **2012**, *3*, 2342. NMR spectra were recorded on a Varian NMR system 500 MHz spectrometer, using D_2O and DMSO-d_6 as the solvents. Gel permeation chromatography (GPC) (against polystyrene standards) was carried out in THF using a Waters system (Waters 1515 pump, Water 2414 refractive 5 index detector) instrument with three styragel HR 0.5, HR 2, HR 4 columns. Transmission electron microscope (TEM) operating at an accelerating voltage of 200 kV was employed for imaging on a Tecnai 20 from FEI. For TEM, the samples were prepared by dropping a solution on the TEM grids and drying under ambient conditions. The particle size was characterized by dynamic light scattering (DLS) at a fixed angle of θ = 165° using an ELSZ-2000 from Otsuka Electronics.

Polymer Synthesis

Synthesis of **PGMA**: glycidyl methacrylate (5 g, 35.17 mmol), 4,4′-dinonyl-2,2′-dipyridyl (dNbpy) (81 mg, 0.20 mmol), ethyl α-bromoisobutyrate (EBiB) (19 mg, 0.1 mmol), and anisole (10 mL) added to a schlenk tube and was purged by bubbling Ar for 30 min. Cu(I)Br (14 mg, 0.1 mmol) was added and Ar purging was continued for another 5 min. The reaction mixture was then stirred under inert atmosphere at room temperature for overnight. After this time, the reaction mixture was cooled to room temperature and precipitated into isopropanol, filtered, and passed through a small plug of silica gel using THF as an eluent. The organic solvent was reduced under low pressure and then precipitated into isopropanol. The obtained white powder was then dried under high vacuum conditions. ^1H NMR (500 MHz, DMSO-d_6) δ 4.30 (br s, COO**CH**$_2$), 3.74 (br s, COO**CH**$_2$), 3.21 (br s, CH_2**CH**CH_2O), 2.80 (br s, $COOCH_2CH$**CH**$_2$O), 2.66 (br s, $COOCH_2CH$**CH**$_2$O), 2.17 – 0.57 (br m, backbone). GPC(THF): M_n = 36400, M_w = 41900, PDI (M_w/M_n) = 1.15.

Synthesis of PEG-*b*-PGMA (**4**): PEG macroinitiator **3** (5kDa) (1 g, 0.2 mmol), glycidyl methacrylate (2 g, 14.1 mmol), 4,4′-dinonyl-2,2′-dipyridyl (dNbpy) (153 mg, 0.37 mmol), and DMSO (3 mL) added to a schlenk tube and was purged by bubbling Ar for 30 min. Cu(I)Br (17.7 mg, 0.12 mmol) was added and Ar purging was continued for another 5 min. The reaction mixture was then stirred under inert atmosphere at room temperature for 2 hrs. After this time, the reaction mixture was cooled to room temperature and precipitated into isopropanol, filtered, and passed through a small plug of silica gel using THF as an eluent. The organic solvent was reduced under low pressure and then precipitated into isopropanol. The obtained white powder was then dried under high vacuum conditions. ^1H NMR (500 MHz, DMSO-d_6) δ 4.28 (br s, COO**CH**$_2$), 3.74 (br

s, COOCH_2), 3.51 (br s, CH_2CH_2O), 3.23 (s, OCH_3), 3.22 (br s, CH_2**CH**CH_2O), 2.80 (br s, $COOCH_2CH$**CH_2**O), 2.66 (br s, $COOCH_2CH$**CH_2**O), 2.20 – 0.66 (br m, backbone). GPC(THF): M_n = 14400, M_w = 17900, PDI (M_w/M_n) = 1.24.

Synthesis of polyseleno-ethers through selenium-epoxy 'click' reaction: To a solution of polymer **PGMA** or **4** (7.04 mmol of epoxy units) and diselenide (3.52 mmol (0.5 eq. per epoxide)) in THF (9 mL) at 0 °C, a solution of sodium borohydride (7.74 mmol (2.2 eq. per diselenide)) in water (1 mL) was added dropwise. The reaction was exothermic and the evolution of hydrogen gas could be observed. The reaction mixture was then stirred at room temperature for 1 hour. After this time, the reaction mixture was precipitated into methanol twice and hexane twice then dried under high vacuum conditions.

Polymer **1**: ^{1}H-NMR (500 MHz, DMSO-d_6) δ 7.45 (br s, -Se**Ph**), 7.21 (br s, -Se**Ph**), 5.34 (br s, CH(**OH**)CH_2Se), 3.88 (br m, COO**CH_2CH**(OH)), 3.02 (br d, CH(OH)**CH_2**Se), 2.17 – 0.57 (br m, backbone). ^{77}Se-NMR (95 MHz, DMSO-d_6) δ 267.9. GPC(THF): M_n = 42500, M_w = 52400, PDI (M_w/M_n) = 1.23.

Polymer **5**: ^{1}H-NMR (500 MHz, DMSO-*d*6) δ 7.45 (br s, -Se**Ph**), 7.21 (br s, -Se**Ph**), 5.33 (CH(**OH**)CH_2Se), 3.89 (br m, COO**CH_2CH**(OH)), 3.51 (br s, **CH_2CH_2**O), 3.23 (s, O**CH_3**), 3.02 (br d, CH(OH)**CH_2**Se), 2.20 – 0.66 (br m, backbone). ^{77}Se-NMR (95 MHz, DMSO-*d*6) δ 267.1. GPC(THF): M_n = 16300, M_w = 20100, PDI (M_w/M_n) = 1.23.

Synthesis of polyselenoniums through alkylation: To a solution of polymer **1** or **5** (50 mg/mL) and $AgBF_4$ (1.25 eq. per seleno-ether units) in MeCN was added alkyl iodide (4 eq. per seleno-ether units) and the reaction mixture was stirred under an inert atmosphere in the dark at 50 °C for 24 hr. After this time, AgI was removed by syringe filter and the filtrate was precipitated into diethyl ether. Polymer was dissolved in MeCN, transferred to a dialysis tube (cutoff 1k Da), and then dialyzed against DI water for 1 day. Polymer solution was lyophilized to dryness to give the product.

Polymer **2**: ^{1}H NMR (500 MHz, DMSO-*d*6) δ 7.90 (br s, -Se^+**Ph**), 7.65 (br s, -Se^+**Ph**), 6.07 (br s, CH(**OH**)CH_2Se), 4.39 – 3.44 (br m, COO**CH_2CH**(OH)**CH_2**Se), 3.03 (br s, -Se^+(**CH3**)), 2.17 – 0.57 (br m, backbone). ^{1}H NMR (500 MHz, D_2O) δ 7.78 (br s, -Se^+**Ph**), 7.55 (br s, -Se^+**Ph**), 4.44 (br m, $COOCH_2$**CH**(OH)CH_2Se), 4.23 – 3.45 (br m, COO**CH_2**CH(OH)**CH_2**Se), 3.04 (br s, -Se^+(**CH_3**)), 2.51 – 0.35 (br m, backbone). ^{77}Se NMR (95 MHz, DMSO-*D*6) δ 382.7, 387.8.

Polymer **6**: ^{1}H-NMR (500 MHz, DMSO-*d*6) δ 7.90 (br s, -Se^+**Ph**), 7.68 (br s, -Se^+**Ph**), 6.07 (br s, CH(**OH**)CH_2Se), 4.39 – 3.55 (br m, COO**CH_2CH**(OH)**CH_2**Se), 3.51 (br s, **CH_2CH_2**O), 3.23 (s, O**CH_3**), 3.07 (br d, -Se^+(**CH_3**)), 2.17 – 0.57 (br m, backbone). ^{77}Se-NMR (95 MHz, DMSO-*d*6) δ 382.2, 381.3.

Dealkylation process: Polymer **2** (7 mg, 0.02 mmol of selenonium units) was dissolved in DI water (1.2 mL) and then added to APDC (4 mg, 0.24 mmol, 1.1 eq. per selenonium unit) solution in DI water (100 μL). The reaction mixture was immediately quenched by liquid nitrogen and then lyophilized to dryness.

Preparation of micelle solution: Polymer **5** (6 mg) was dissolved in THF (300 μL), added dropwise into deionized (DI) water (6 mL) under stirring, and then filtered through a 0.2 μm syringe filter (cellulose acetate). The mixture was dialyzed (dialysis tube: cutoff 1 kDa) against deionized water for 2 days to remove THF. The final polymer concentration was about 1 mg/mL in water.

Micellar disassembly process: $AgBF_4$ (1.25 eq. per seleno-ether units) and iodomethane (4 eq. per seleno-ether units) were added to a micelle solution (5 mL) and the reaction mixture was stirred at 50 °C for 24 hr. After this time, AgI was removed by syringe filter and the filtrate dialyzed (dialysis tube: cutoff 1 kDa) against deionized water for 1 day to remove residual reagents.

Micellar assembly process: APDC (1.1 eq. per seleno-ether units) was added to 5 mL of aqueous solution obtained upon polymer disassembly process. As soon as the APDC added, the solution turned cloudy and quenched by liquid nitrogen then lyophilized to dryness.

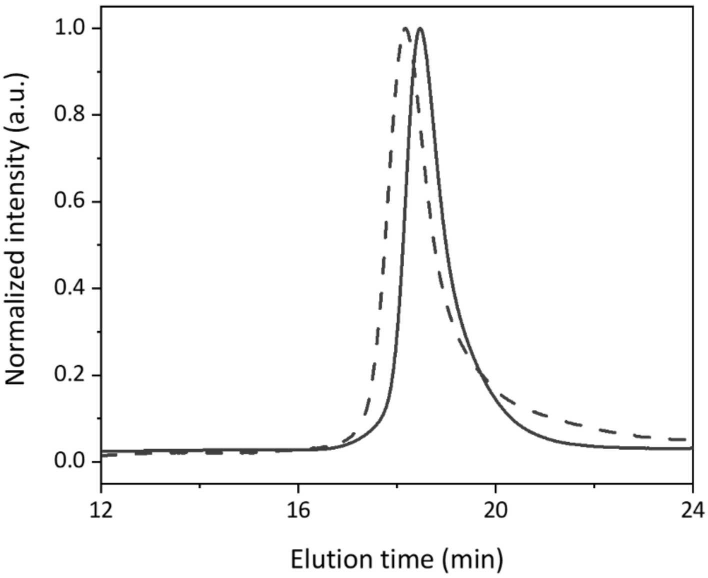

Figure S1. GPC of **PGMA** (solid line) and polymer **1** (dash line).

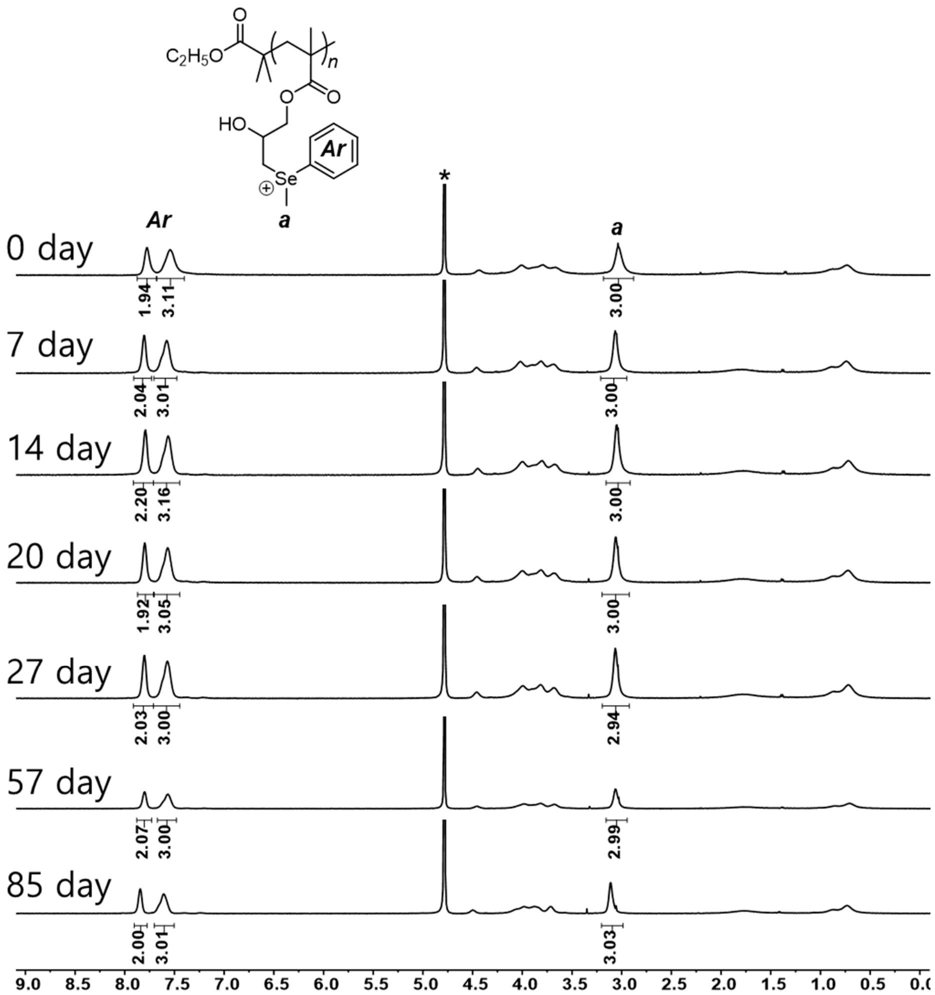

Figure S2. ^{1}H-NMR of Polymer **2** in D_2O at 37 °C. Residual solvent signal is shown with an asterisk.

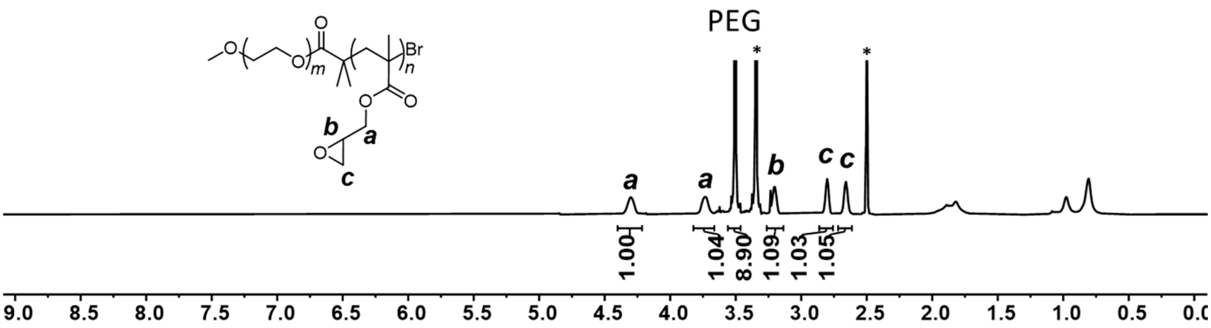

Figure S3. ¹H-NMR of polymer **4** in deuterated dimethylsulfoxide (DMSO). Residual solvent signal are shown with an asterisk.

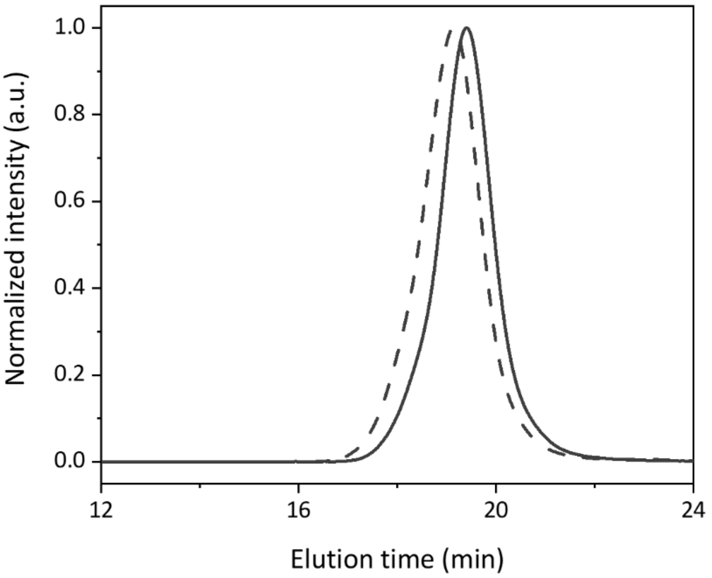

Figure S4. GPC of polymers **4** (solid line) **5** (dash line).

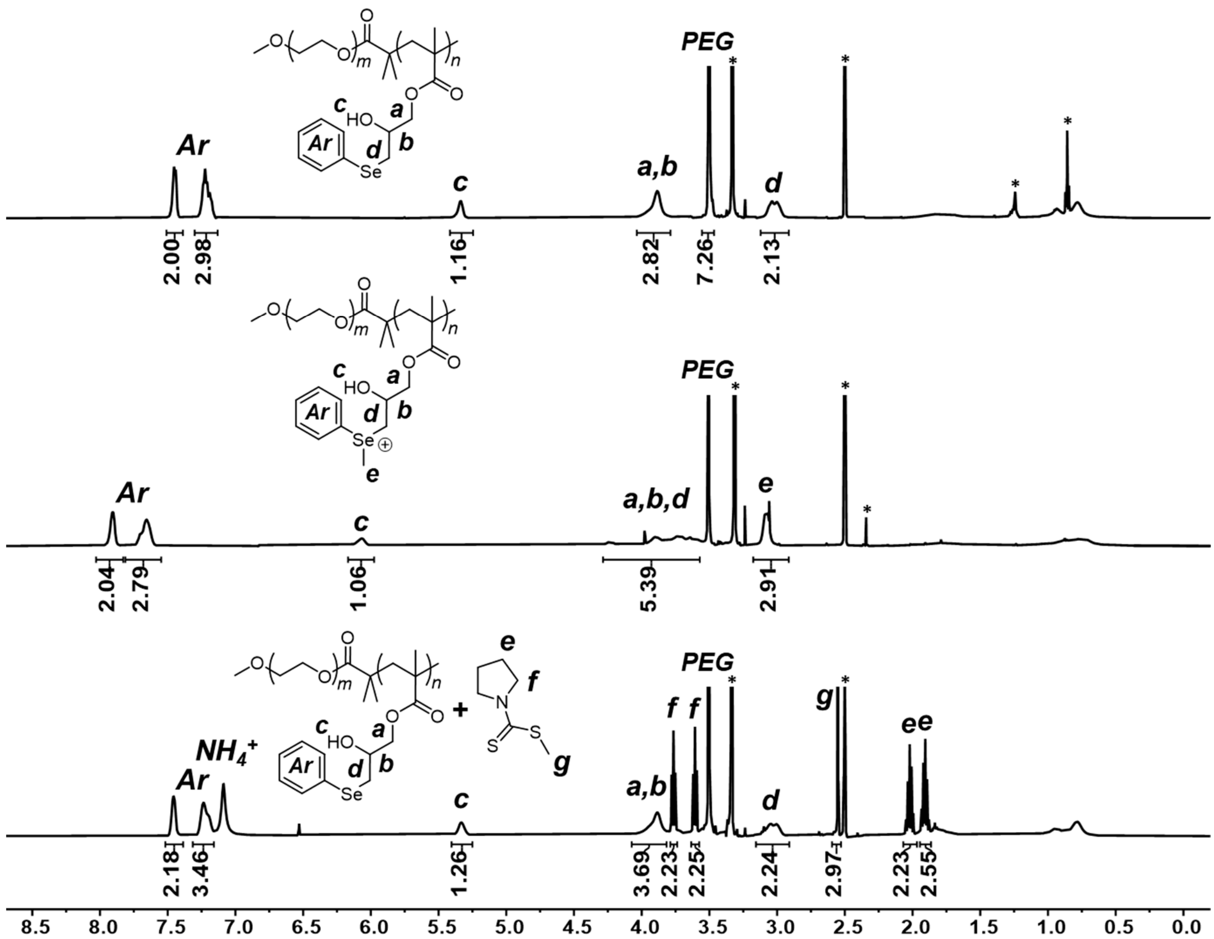

Figure S5. ^{1}H-NMR of polymer **5** (top), after alkylation (middle), and upon exposure with APDC (bottom). Signals from residual solvents are shown with an asterisk. The measurements were carried out immediately after addition of APDC to the polymer solution.

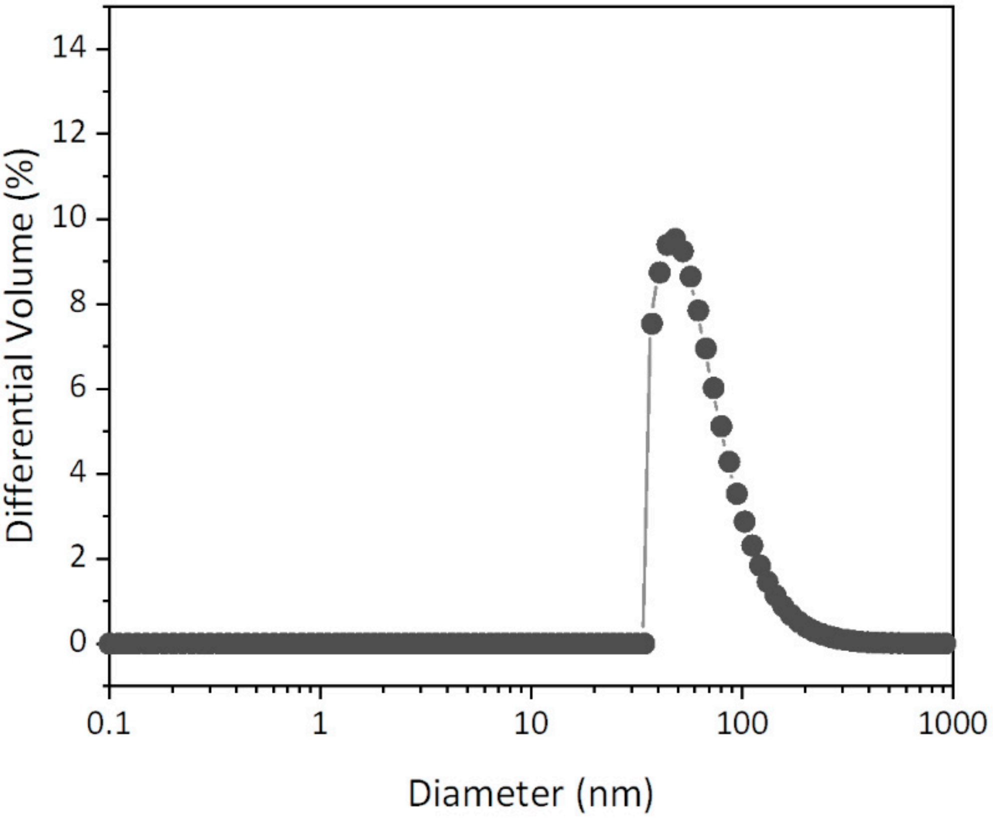

Figure S6. DLS data for the control experiment in which the micelles of block copolymer **5** were exposed to 50 °C in absence of silver tetrafluoroborate and methyl iodide for 24 hours prior to data acquisition (diameter = 63.1 nm, PDI = 0.196).

Table S1. DLS data for polymer **5** before and after dealkylation process.

	**Solvent**	**Diameter (nm)**	**PDI**
Polymer **5**	Water	62.3	0.183
Polymer **5** after alkylation	Water	0.1	-
Polymer **5** after dealkylation	Water	53.3	0.191
